# Ubiquitylation in ERAD: Reversing to Go Forward?

**DOI:** 10.1371/journal.pbio.1001038

**Published:** 2011-03-29

**Authors:** Yien Che Tsai, Allan M. Weissman

**Affiliations:** Laboratory of Protein Dynamics and Signaling, National Cancer Institute, Frederick, Maryland, United States of America

## Summary

Proteins are co-translationally inserted into the endoplasmic reticulum (ER) where they undergo maturation. Homeostasis in the ER requires a highly sensitive and selective means of quality control. This occurs through ER-associated degradation (ERAD). This complex ubiquitin-proteasome–mediated process involves ubiquitin conjugating enzymes (E2) and ubiquitin ligases (E3), lumenal and cytosolic chaperones, and other proteins, including the AAA ATPase p97 (VCP; Cdc48 in yeast). Probing of processes involving proteasomal degradation has generally depended on proteasome inhibitors or knockdown of specific E2s or E3s. In this issue of *PLoS Biology*, Ernst et al. demonstrate the utility of expressing the catalytic domain of a viral deubiquitylating enzyme to probe the ubiquitin system. Convincing evidence is provided that deubiquitylation is integral to dislocation of ERAD substrates from the ER membrane. The implications of this work for understanding ERAD and the potential of expressing deubiquitylating enzyme domains for studying ubiquitin-mediated processes are discussed.

## The Endoplasmic Reticulum, Protein Synthesis, and Degradation

The membranous network that constitutes the endoplasmic reticulum (ER) plays essential roles in all metabolically active eukaryotic cells. The ER is contiguous with the nuclear envelope, yet it extends throughout the cytoplasm contacting mitochondria and coming in close proximity to the plasma membrane. This organelle plays important roles in cell metabolism, regulation of apoptosis, signal integration via calcium signaling, and sensing of the cell's microenvironment. Importantly, the ER is the port of entry for the vast majority of newly synthesized proteins that traverse the interconnected secretory and endocytic pathways. Proteins that are not normally occupants of the ER are transported by vesicular trafficking to the Golgi network. From there, these proteins reach their ultimate destinations as resident proteins of the secretory or endocytic pathways or the plasma membrane; alternatively, they are secreted from the cell. Proteins that enter the ER and traverse these pathways can constitute up to a third of synthesized proteins. It is therefore essential that the functional status of the ER be continually monitored.

In general, proteins are co-translationally inserted into the ER through a narrow aqueous channel known as the Sec61 translocon [1]. This is coupled to early steps in protein maturation that can include the addition of N-linked oligosaccharides, which undergo further modification in the Golgi network, and complex chaperone-mediated folding that is often integrally associated with the formation of highly specific disulfide bonds [2],[3]. In many cases, these newly synthesized proteins also begin the process of assembly into complexes that ultimately result in functional receptors or channels. This requires that components “find each other” in the ER and oligomerize in a highly specific and ordered manner through interactions involving domains in the lumen of the ER and the ER membrane as well as their cytoplasmic domains.

The complexity of the processes involved in protein folding and assembly and the need to regulate levels of critical resident proteins of the ER, such as HMG CoA reductase, requires the ER to have efficient and regulated means to dispose of unwanted proteins. Thus, much effort was expended in the 1980s and early ′90s towards identifying a proteolytic system in the ER or another pre-Golgi compartment using models including the pre-Golgi degradation of unassembled components of the T cell antigen receptor (TCR) [4]. Eventually it became apparent that degradation of these unassembled subunits as well as other proteins does not occur within the secretory pathway. Instead, ER proteins are targeted for export to the cytosol where they are degraded, much as their cytosolic counterparts, by the ubiquitin-proteasome system (UPS) [5]–[9]. This takes place through a coordinated process known as ER-associated degradation (ERAD) [3],[10],[11] (*vide infra*).

## ER Stress and the Unfolded Protein Response

Imbalance between neo-synthesis, degradation, and transport through the secretory pathway results in “ER stress” that, if left uncompensated, threatens cell function and survival and is linked to numerous pathologies, including neurodegenerative diseases, cancer, and diabetes [12]. In higher eukaryotes, the state of the ER is continually monitored by at least three ER transmembrane sensors that each initiate a set of distinct but intersecting signaling pathways oriented towards maintaining ER homeostasis. This graded response, which is dependent on the degree of ER stress, is collectively known as the unfolded protein response (UPR) [13],[14]. The UPR upregulates the expression of genes involved in protein folding, modification, transport, and degradation, as well as redox regulation. The UPR also results in an increase in expression of enzymes for lipid biosynthesis, so as to increase the surface area of the ER. At the same time, the UPR inhibits global protein translation and reduces protein translation at the ER to reduce the protein load of the ER [15]. This integrated response allows cells to conserve resources and overcome ER stress. If the UPR fails to restore ER homeostasis, cell death pathways are activated. One important mechanism for alleviating ER stress is by increasing degradation from the ER through UPR-mediated upregulation of ERAD components. Conversely, loss of ERAD components sensitizes cells to ER stress–induced apoptosis. The quality control/ERAD system becomes even more important during cellular stress, when small changes in the cell's ability to cope with ER stress can tip the balance towards either death or survival and proliferation [11].

## Endoplasmic Reticulum–Associated Degradation

**Figure 1:**
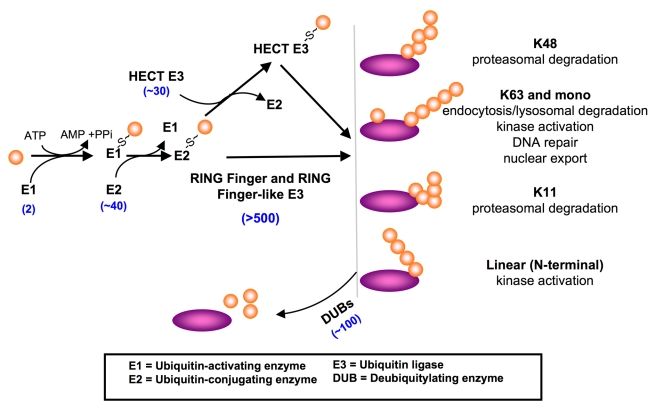
Roles of ubiquitylation in cellular regulation. Conjugation of ubiquitin onto protein substrates requires at least three enzymes. One of two ubiquitin activating enzymes (E1) activates ubiquitin through an ATP-dependent step, forming a thioester linkage between the active site cysteine of E1 and the C-terminal carboxylate of ubiquitin. E1 then transfers the ubiquitin to the active site cysteine of one of approximately 40 mammalian ubiquitin conjugating enzymes (E2). The ubiquitin can be transferred to the active site of a HECT domain ubiquitin ligase (E3), which binds the substrate and mediates the conjugation of ubiquitin. RING finger and related E3s function as allosteric activators of E2, promoting the transfer of ubiquitin directly from the E2 to the substrate. The combination of E2/E3 determines the length and type of polyubiquitin chains assembled on the substrate, which can lead to diverse cellular effects, some of which are noted. Ubiquitylation is best characterized as modifying primary amines (lysines and the N-termini of proteins) [44]. More recently, there has been evidence that other nucleophillic amino acids including serine, threonine, and cysteine can also be modified with polyubiquitin chains [45]–[48]. DUBs perform a number of cellular roles, including removing ubiquitin from specific substrates. There are at least five classes of DUBs, and many DUBs show strong preferences for specific polyubiquitin chain linkages.

We now understand that ERAD involves substrate ubiquitylation and proteasomal degradation, which are tightly coupled to substrate dislocation from the ER. The conjugation of proteins with ubiquitin is a highly regulated process with specificity conferred by over 500 different ubiquitin ligases (E3s) working together with ubiquitin conjugating enzymes (E2s). The specific consequence of ubiquitylation is largely dependent on the nature of the polyubiquitin chain formed on target proteins. Besides proteasomal degradation, modification of proteins with single ubiquitin or polyubiquitin chains, commonly linked through different lysines of ubiquitin, can have other proteasome-independent functions in DNA repair, NF-κB activation, endocytosis, and lysosomal targeting, as well as other processes [16]. Ubiquitylation is opposed by the action of ∼100 mammalian deubiquitylating enzymes (DUBs) [17]. DUBs perform many functions, including the deubiquitylation of specific substrates and disassembly of specific ubiquitin linkages (Figure 1).

The ERAD ubiquitylation machinery in both yeast and mammals consists primarily of ER-resident transmembrane E3s and their cognate E2s. In several cases, particularly in mammals, multiple E3s are implicated in the degradation of a specific substrate. DUBs can counteract the activity of ubiquitin ligases towards specific ERAD substrates [18], although the roles of DUBs in ERAD have not been extensively explored.

Intense interest has been focused on identification of the protein conducting channel(s) through which misfolded proteins can be exported from the ER to the cytosol—a process variously referred to as “retrotranslocation” or “dislocation.” Early studies suggested that Sec61, the import channel for proteins into the ER, might also be the retrotranslocon through which this dislocation is effected [8],[19]–[21]. Another such candidate is Derlin-1, a polytopic protein implicated in the targeting of several substrates for degradation [22],[23]. In addition, polytopic ER-resident ubiquitin ligases such as Hrd1 have been suggested to form part of this channel [24]. As Sec61 imports nascent polypeptides into the ER in an unfolded state, a reasonable assumption is that protein unfolding is likely required for retrotranslocation. However, it is not evident that this is uniformly the case [25],[26].

**Figure 2:**
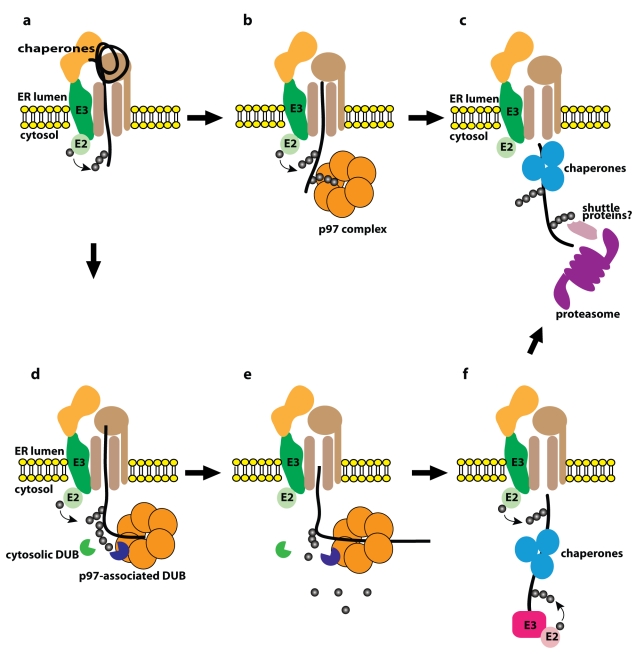
Models for ERAD. (a–c) Classical view of ERAD. (a) ERAD substrate (black) is recognized by ER chaperones and partially translocated through a protein conducting channel complex/retrotranslocon (brown). The substrate is conjugated with chains of ubiquitin by an ER-resident ubiquitin ligase (E3) and its cognate ubiquitin conjugating enzyme (E2) on the cytosolic face of the ER membrane. (b) The p97 complex, comprising a hexamer of the AAA ATPase p97 and accessory proteins such as Ufd1 and Npl4 (not depicted), associates with the retrotranslocation complex, recognizes the polyubiquitin chain and extracts the ubiquitylated substrate to the cytosol. (c) Polyubiquitin chains target the dislocated substrate to the 26S proteasome (magenta) for degradation, in some cases assisted by shuttle proteins (pink) that bind both to ubiquitin chains and the proteasome. (d–f) Model based on results in Ernst et al. [33],[36]. The exact mechanism by which the p97 complex extracts the substrate is not well understood. These new findings suggest that (d) the p97 complex recognizes polyubiquitin chains on the substrate as it moves through the protein-conducting channel. (e) DUBs associated with the p97 complex (purple) or potentially free in the cytosol (green) trim off the polyubiquitin chain on the substrate, allowing it to be threaded into the narrow channel of the p97 complex. (f) The dislocated substrate is ubiquitylated a second time by either ER-resident or cytosolic E3s for targeting to the proteasome (depicted in [c]).

For transmembrane proteins with cytosolic domains, the topological conundrum of being ubiquitylated and degraded from the ER is easily conceptualized. Exposed domains of substrates serve as targets for the ubiquitylation machinery associated with retrotranslocons (Figure 2). Ubiquitylated species are “ratcheted” out of the ER in an ATP-driven process provided by complexes of the hexameric AAA-ATPase p97 (Cdc48 in yeast) and associated ubiquitin-binding proteins [27],[28]; these complexes are also implicated as intermediates in the proteasomal targeting of non-ER proteins [29]. At least in yeast, there is evidence that other ubiquitin-binding “shuttle proteins” play a role in delivering dislocated substrates from p97 complexes to proteasomes [30]. For those proteins that reside in the lumen of the ER, the means by which they are retrotranslocated is conceptually more difficult to envision. However, we now understand that lumenal chaperones can associate with at least one of the transmembrane ubiquitin ligase complexes and deliver proteins to sites of ubiquitylation [31],[32].

## Deubiquitylation as an Integral Step in ERAD

A recent study by Ernst and colleagues has provided a new and interesting twist to ERAD [33]. The p97 complex has been found to be associated with several deubiquitylating enzymes, including YOD1 and USP13 [34]. YOD1 interacts with p97 via its UBX domain. Transfection of a catalytically inactive form of YOD1 blocks dislocation of model ERAD substrates [33]. This result suggests that deubiquitylation is required for protein dislocation from the ER (Figure 2). This is a finding that at first seems counterintuitive, given the wealth of evidence suggesting a role for ubiquitylation in this process. One possibility is that, analogous to threading of the substrate into the narrow channel of the proteasome, protein dislocation might require trimming off the polyubiquitin chain to allow the substrate to enter the central channel of the p97 complex during retrotranslocation. While highly provocative, these results involve overexpression of a “dominant negative” form of YOD1. This overexpression could potentially interfere with the binding to p97 of other important UBX domain-containing components of the ERAD machinery [35]. Thus, the possibility that the results obtained were an indirect effect of disrupting other p97 interactions could not be discounted.

Until now, with few exceptions, probing of the UPS in general and ERAD in particular has relied on either manipulation of substrate-specific enzymes of the ubiquitin system or use of proteasome inhibitors. In the current issue, Ernst and colleagues [36] unveil a new tool for inhibiting protein degradation—the catalytic domain of a DUB encoded by the Epstein-Barr virus (EBV). The authors show that the isolated EBV DUB domain inhibits degradation of proteasome substrates, likely due to preemptive removal of ubiquitin from the substrates. Interestingly, cells tolerate expression of the EBV DUB better than proteasome inhibitors. They then employ this tool to follow up on the aforementioned study on the role of DUB activity in protein dislocation.

By fusing the catalytic domain of EBV DUB with a UBX domain, Ernst et al. target the EBV DUB domain to p97-containing complexes. Expression of this UBX-EBV DUB also results in accumulation of ERAD substrates. For two model substrates, a mutant ribophorin RI_332_ and TCRα, accumulation of deglycosylated intermediates suggest that these proteins are largely dislocated, since N-glycanase, which is responsible for removing N-linked oligosaccharides from proteins, is confined to the cytosol. Subcellular fractionation and microscopy confirm that the substrates are dislocated but remain loosely associated with the ER membrane. The authors then take advantage of this p97-targeted DUB to further probe the role of ubiquitylation during ERAD. Strikingly, expression of UBX-EBV DUB restores protein dislocation in the presence of catalytically inactive YOD1. The dislocated substrates accumulate in the cytosol as deglycosylated and deubiquitylated intermediates. These results reinforce the idea that deubiquitylation is necessary for substrate dislocation. However, as the EBV DUB overcomes the inhibitory effect of inactive YOD1 even when not targeted to p97 via a UBX domain, it leaves unanswered whether, for endogenous DUBs, p97 association is truly required for their recruitment to the ER and function in ERAD. It also remains to be formally established whether the critical targets for this deubiquitylation step are the ERAD substrates, although this is certainly the most likely scenario. Such a model would then require at least two rounds of ubiquitylation to occur during ERAD. The first round is essential for p97 recognition and dislocation. The second round, following substrate deubiquitylation, is essential for proteasomal targeting of the now dislocated protein.

## Cytosolic Chaperones in ERAD

As proteins dislocated from the ER may be partially unfolded, an important question is how they are maintained in a soluble form in the cytosol. Previous studies have shown that cytosolic chaperones are involved in the degradation of certain ERAD substrates [37]–[40]. Employing chemical inhibitors or mutants of the dislocation machineries, Ernst et al. utilized mass spectrometry to identify proteins interacting with a model ERAD substrate, RI_332_, when protein dislocation is disrupted at different stages. This approach should in principle allow for a systematic and unbiased discovery of factors involved in the various stages of ERAD. For example, expression of UBX-EBV DUB or treatment with proteasome inhibitors arrests some percentage of the substrate at a step after dislocation. Under these conditions, RI_332_ associates with cytosolic chaperones, supporting the idea that a cytosolic chaperone network buffers the dislocated substrate and allows their subsequent degradation by the proteasome. The role of these cytosolic chaperones in ERAD awaits further studies.

## Future Directions

These recent findings raise new questions regarding ERAD and illustrate the utility of DUBs in studying the UPS. If substrate deubiquitylation is required for retro-translocation, an important question now becomes what components of UPS participate in the subsequent proteasomal-targeting step of dislocated substrates. As dislocated substrates stay loosely associated with the ER membrane, it would be interesting to determine whether this second round of ubiquitylation is carried out by the same ERAD ubiquitin ligases that initiate dislocation, or whether cytosolic ubiquitin ligases now assume control. If cytosolic ubiquitin ligases are involved, are they specialized for dislocated substrates or do they overlap with those implicated in cytosolic quality control? In this regard, it is of note that HSP70-associated E3s including Parkin and CHIP have been implicated in the degradation of some ERAD substrates [41],[42].

A major point accentuated by the current findings of Ernst et al. [36] is that DUB catalytic domains can be used to inhibit and probe the ubiquitin conjugating system in ways that might not be achieved by proteasome inhibitors. While the use of such domains can provide important new insights, interpretation of results obtained using these tools have important caveats. These include the fact that, aside from subcellular localization, outcome of expressing a DUB domain will likely be highly dependent on its relative in vivo specificity for different ubiquitin linkages. The specificity of DUBs for different polyubiquitin chain linkages varies considerably. Therefore, results obtained with expression of particular DUBs will need to be interpreted in this context.

Another attractive means of utilizing DUB catalytic domains is to covalently fuse them with proteins that have restricted subcellular localization to probe specific functions of ubiquitylation in the cell. An isolated DUB catalytic domain can also be fused to the target protein to inhibit ubiquitylation of the fusion protein. This strategy has recently allowed the dissection of the role of ubiquitylation in endosomal targeting in yeast [43]. This approach, as well as variations on the one employed in this issue, opens up exciting new possibilities for probing the function of ubiquitylation in cellular regulation.

## Abbreviations

DUB: deubiquitylating enzyme
EBV: Epstein-Barr virus
ER: endoplasmic reticulum
ERAD: ER-associated degradation
TCR: T cell antigen receptor
UPR: unfolded protein response
UPS: ubiquitin-proteasome system
